# Periodontitis Risk Assessment and Prevention Planning: Comparative Study of Multimodal Large Language Models and Periodontist Evaluations

**DOI:** 10.2196/87781

**Published:** 2026-10-02

**Authors:** Laura Swinckels, Katharina Alves Rabelo, Eduardo L Delamare, Bruno G Loos, Pierre Lahoud, Harmen Bijwaard, Ander de Keijzer, Jinman Kim, Josef Bruers

**Affiliations:** 1 Department of Oral Public Health, Academic Center for Dentistry Amsterdam, Gustav Mahlerplein 3004, Amsterdam, North Holland, 1182 DB, The Netherlands, 31 020 59 80888; 2 Cluster of Health, Sport and Welfare, Inholland University of Applied Sciences, Amsterdam, North Holland, The Netherlands; 3 Sydney Dental School, Faculty of Medicine and Health, The University of Sydney, Sydney, New South Wales, Australia; 4 Department of Periodontology, Academic Center for Dentistry Amsterdam, Amsterdam, North Holland, The Netherlands; 5 Division of Periodontology & Oral Microbiology, Department of Oral Health Sciences, University Hospitals Leuven, KU Leuven, Leuven, Flanders, Belgium; 6 OMFS-IMPATH Research Group, Department of Imaging and Pathology, Universitair Ziekenhuis Leuven, Leuven, Flanders, Belgium; 7 Department of Conservative Dentistry, Periodontology and Digital Dentistry, LMU University Hospital, Ludwig-Maximilians-Universität München, Munich, Bavaria, Germany; 8 National Institute for Public Health and the Environment, Bilthoven, Utrecht, The Netherlands; 9 Applied Responsible Artificial Intelligence Research Group, Avans University of Applied Sciences, Breda, North Brabant, The Netherlands; 10 School of Computer Science, Faculty of Engineering, The University of Sydney, Sydney, New South Wales, Australia; 11 Royal Dutch Society for the Promotion of Dentistry, Utrecht, Utrecht, The Netherlands

**Keywords:** large language model, multimodal AI, generative AI, periodontitis, clinical decision support, radiographs, electronic health records, benchmarking, clinical specialist

## Abstract

**Background:**

Periodontitis is one of the most prevalent yet preventable oral diseases, as indicated by multiple clinical and radiographic factors. As these factors are recorded in electronic health records (EHRs), their reuse offers opportunities for personalized risk assessment and targeted prevention. Predictive AI and traditional machine learning models support fragmented detection tasks but lack the integration of textual and imaging predictors. Emerging multimodal large language models (M-LLMs) show promise in combining these data sources for clinical assessment. Evaluating the capabilities of M-LLMs and comparing them against the current clinical standard are therefore essential to determine their potential as digital assistants.

**Objective:**

This study aimed to evaluate the ability of M-LLMs to assess periodontitis risk and suggest prevention strategies, based on EHR data and radiographic findings. Each M-LLM was individually evaluated by periodontal experts, benchmarked against other models, and compared with a periodontist as a reference.

**Methods:**

A vignette study was conducted following TRIPOD (Transparent Reporting of a Multivariable Prediction Model for Individual Prognosis or Diagnosis) guidelines for the evaluation of LLMs. Ten periodontal vignettes were created, each including a panoramic radiograph and textual EHR data. Three LLMs capable of reasoning and handling multimodal data were compared to a periodontist who generated outputs manually, based on the same prompts and input data. Periodontal experts rated all outputs across 6 predefined criteria on a 5-point Likert scale. Statistical analyses evaluated overall performance per model and tested whether performance varied per model, scenario complexity, or rater.

**Results:**

GPT o1 Pro and Claude Sonnet 4 showed strong performance, with 86.7% and 85.6% of ratings deemed acceptable—comparable to the periodontist’s output (87.8%). Gemini 2.5 Pro was rated significantly lower than both the periodontist and the other models (59.4% acceptable; *P*<.002). Radiographic interpretation consistently received lower scores than other abilities across all models and the periodontist, with Gemini rated below the acceptable threshold. The time required for completion ranged from approximately 10 seconds for Claude to 37 seconds for Gemini; 3 minutes, 22 seconds, for GPT; and 5 minutes, 57 seconds, for the periodontist.

**Conclusions:**

M-LLMs demonstrated strong reasoning abilities in periodontal assessment. Across all models, unacceptable elements were consistently related to errors in radiographic interpretation, though refined prompting or newer model versions may improve this. Notably, even when radiographic findings were incorrect and plaque-retentive factors were absent, outputs were still rated well, indicating that EHR data alone provide a substantial basis. For clinical applicability, M-LLMs must at least perform comparably to a periodontist and meet the quality standards set by periodontal experts—a bar that GPT and Claude appear to approach.

## Introduction

### Background

Periodontitis is an inflammatory disease of the periodontal tissues and alveolar bone [1,2], with potential consequences for systemic health [3]. It is among the most prevalent oral diseases [4], despite being largely preventable [5]. As periodontitis is a multifactorial condition [6-8], its assessment and prevention strategies must also be multifactorial [9]. Dental professionals screen for several early clinical indicators (eg, plaque, gingival bleeding, and pocket depth), promote healthy behavior (eg, toothbrushing, interdental cleaning, reducing smoking and alcohol intake), and consider multiple nonmodifiable risk factors such as age, sex, hormonal changes, diabetes, cardiovascular disease, and immunodeficiencies [10]. These factors are typically evaluated during routine checkups and should be documented in electronic health records (EHRs) [10-13]. In addition, radiographs are used to assess alveolar bone levels and plaque-retentive factors, such as calculus, defective restorative margins, and interdental caries, affecting periodontal activity [10,14-16]. Given the large number of influencing factors, periodontitis cannot be treated with a one-size-fits-all approach but requires precise and personalized strategies for prevention and management [17].

All information relevant to a patient’s dental care (ie, aforementioned factors) should be recorded in EHR systems [11]. Because dental patients visit their dental professionals regularly, a large amount of data accumulate over time and continue to grow throughout their lifespan, resulting in extensive records [18]. Ideally, this information should be reused; however, in the fast-paced setting of daily dental practice, it is often time-consuming to retrieve and integrate all factors [19], particularly when combining textual EHRs and imaging-derived data. When such information is not or only partially considered, clinical decisions become generalized rather than personalized, insufficiently substantiated, and may lead to over- or undertreatment. Digital assistance could therefore help streamline data-driven decision-making and improve consistency across dental professionals.

AI has been widely explored as a potential solution to support (preventive) clinical decisions [20,21]. Most current dental applications are “narrow” models, developed for specific closed tasks with probabilistic yes/no outputs. For example, deep learning computer vision models can detect alveolar bone loss (ABL) or plaque-retentive factors on radiographs [22-24]. While useful, these approaches are limited to single dental outcomes and still require integration with other relevant data for a comprehensive assessment. More traditional machine learning techniques can estimate periodontal risk by combining multiple textual factors from EHR [8,25], but still lack the required imaging findings. Therefore, AI support is fragmented and a complete periodontal assessment still relies on the dental professional’s effort to combine and comprehensively translate these AI findings.

Generative AI, particularly large language models (LLMs), offers broader potential by generating new content for open-ended tasks (eg, explanations and recommendations) [26]. Literature reviews across several medical domains highlight how LLMs can interpret medical results, write reports, support clinical reasoning, and guide follow-up care [27-30]. In dentistry, LLMs such as GPT-4 have a pooled accuracy of 73% on dental licensing exams [27]. However, recognizing or recalling knowledge represents a lower cognitive level, while applying theory to complex scenarios is essential for real-life clinical reasoning and dental competence. Specifically related to periodontal knowledge, LLMs were able to answer most exam questions [31-33], but the integration of radiographic data requires further improvement [34]. Newer, more advanced model versions are being launched at a high pace [35]. Recent versions called “reasoning models” apply “chain-of-thought” prompting to already established LLMs [36]. These reasoning models extend in their multistep problem solving, scientific reasoning, and workflow planning [37]. Additionally, LLM providers now claim that some versions can process multimodal input (including text, images, sound, or video) [38,39], bringing these forms of AI closer to the integrative analyses required in dentistry.

Multimodal LLMs (M-LLMs) are designed to integrate and interpret complex datasets from multiple modalities on which clinical decisions in health care often rely [38]. These LLMs handle input and output across different modalities (eg, text-to-image or image-to-text conversions) or process text and images simultaneously. Early work in 2023 showed that text-only LLMs performed poorly on image interpretation tasks in oral disease cases (accuracies <35%) [40]. Later multimodal approaches have shown notably better results with newer models. GPT-4o achieved 71.4% accuracy in identifying lesion location, 58.2% in diagnosing oral mucosal lesions from clinical photographs, and, when the diagnosis was correct, over 90% accuracy in recommending diagnostic tests and treatments [41]. In oral pathology, the multimodal GPT-4o reached the highest accuracy in diagnosing oral premalignant lesions when text and image data were combined, outperforming GPT-4.0, GPT-3.5, and Gemini [42]. Interestingly, one study found that a text-only LLM (DeepSeek) outperformed a multimodal model in complex oral disease cases, particularly high-difficulty or inflammatory lesions [43], suggesting that contextual clinical information may sometimes be more critical than imaging alone. These findings, to date, indicate that the current M-LLMs show potential for including image findings, but textual and contextual data remain essential.

Notably, most models have been tested on closed or multiple-choice questions rather than on open-ended case assessments, underscoring the need to evaluate their performance in realistic clinical scenarios. It is important to benchmark the currently available models, rather than waiting for “perfect” versions, as clinicians and patients are already likely to access them. Major EHR providers have already started the integration of LLMs into medical software in 2023 [44], so clinical use is only a matter of time. Understanding both the capabilities and limitations of these off-the-shelf models is therefore essential for safe, informed, and desired use in practice.

### Study Objectives

This study aimed to evaluate the ability of M-LLMs to assess the risk of developing or progressing periodontitis, based on EHR data and radiological findings. Each M-LLM’s output was rated individually by periodontal experts, benchmarked against those of other models, and compared to a reference standard: a human-generated periodontal risk assessment.

## Methods

### Ethical Considerations

This study was conducted in accordance with the TRIPOD-LLM (Transparent Reporting of a Multivariable Prediction Model for Individual Prognosis or Diagnosis-Large Language Models) reporting guideline for studies using LLMs [45], specifically to evaluate LLMs. A vignette research design was employed [46] to evaluate the AI-generated periodontal assessments and to compare them to human-generated periodontal assessments, with all outputs independently reviewed by 3 periodontal experts (serving as the expert standard). These experts were informed and provided consent to participate in this study without financial compensation. The institutional ethics committee of the Academic Center for Dentistry Amsterdam approved the study under the number 2025‐31346. Data were anonymized by reusing real-life orthopantomograms (OPGs) that were approved for research purposes, complemented with synthetic EHR data, to ensure privacy and confidentiality. The methods will be described in 4 evidence-based steps.

### Periodontal Vignettes

Ten scenarios are described in vignettes that mimic frequent and well-known clinical cases in periodontology (Multimedia Appendix 1). Each scenario includes (textual) EHR data and an OPG, which should ideally be included to assess the periodontal risk of each patient [47]. Scenarios included healthy individuals and patients with gingivitis and periodontitis. OPGs were selected from a public dataset, DENTEX [48], that corresponds with the scenarios. This public dataset enables the reuse of dental radiographs for evaluation and benchmarking purposes. As this study focuses on prevention, the majority of OPGs (6/10) show normal alveolar bone levels, and a minority of OPGs show mild to advanced ABL (4/10) to test their ability to differentiate between periodontitis cases and controls. Among the OPGs without marginal ABL, a diversity of plaque-retentive risk factors was selected, including calculus, interproximal carious lesions, and defective restorative margins, which may affect the periodontal risk. An oral radiologist and a dental hygienist who did not participate as evaluators selected the OPGs for each scenario.

In addition to the OPG selection, each scenario includes textual clinical indicators, lifestyle factors, and nonmodifiable patient-specific factors. These input data were simulated in a format that could have been extracted from EHR systems. Creating simulated scenarios instead of using real patient records safeguarded privacy, which is highly important for publicly available LLMs, as real EHR data combined with corresponding OPGs cannot be assumed to be fully deidentifiable. Clinical indicators included a periodontal screening index, bleeding-on-probing percentage, and plaque percentage, and they adhered to the most recent or internationally applied recording standards (eg, Periodontal Screening and Recording Index (PSI) [49] and Bleeding on Probing percentage [50,51]). Lifestyle factors, including oral hygiene features, are often documented as free-text clinical notes and were therefore written in a succinct manner. Smoking was dichotomously recorded. Medical conditions were recorded at the *International Classification of Diseases* (*ICD*) group level [52] to ensure diseases were consistently grouped. As dental EHRs often contain missing data, we deliberately included this in our scenarios to reflect real-world documentation practices. For instance, although plaque and bleeding on probing percentages are valuable indicators, they are frequently omitted when the PSI is low.

Each scenario was double-checked to avoid contradicting the EHR data and imaging findings. When the scenarios were complete, they were reviewed by a periodontist, and after minor modifications, they were provided as input data to the model.

### The Selection of M-LLMs

Multiple M-LLMs were comprehensively considered: GPT o3, o1 Pro, Gemini 2.0 Flash and 2.5 Pro, Claude Sonnet 4, LlamaMed++, LlaVa Med, Qwen, Grok, and Copilot. M-LLMs must have reasoning ability (which is suitable for medical cases) and must be able to handle both textual and imaging input data. The selection of 3 models was based on publications in the dental literature, model features as of July 2025 [53-55], and our experience during the pilot. However, the existing literature often lacks up-to-date information on the latest model versions, resulting in a lack of published results on the most recent and advanced models. Most models were excluded due to their limited ability to reason, the number of parameters, their computational power (like CPU/GPU), and the quality of pilot results. The selected M-LLMs are publicly available and run on a high-capacity infrastructure, allowing access to large, multimodal models with superior performance. Although local models have the advantage of protecting patient privacy, we did not choose them because they lag behind in scale and accuracy on complex tasks by the time of this study. The models selected are currently being integrated into health care settings and represent the models most widely used by both patients and clinicians in practice [56].

GPT has a collection of models, with only a few having reasoning abilities. Mini versions of GPT were excluded due to their limited capacity, which restricts their ability to process multimodal data. Only GPT o1 Pro and GPT o3 were full-capacity models (ie, no “mini” versions) with reasoning abilities, allowing both text and image input data. At the time of this research, these models were novel and had not yet been scientifically evaluated in dentistry and were claimed to have specific features by their manufacturer, OpenAI. No direct comparative evidence between GPT o3 and GPT o1 Pro in multimodal settings was available in the literature. Based on the pilots conducted, we found that GPT o1 Pro included more radiographic findings in its output (regardless of their correctness) than GPT o3, so we expected that this model would have a slightly better ability to analyze radiographs or at least to extract radiographic findings. The newest GPT-5 model was rolled out just after data collection had started. The GPT versions on a specific date (compared to the general model) were recommended for research and reproducibility; thus, we included GPT o1 Pro (end point identifier: o1-pro; snapshot dated March 19, 2025) in this study [54].

Gemini 2.5 Pro was also assumed to have “enhanced reasoning” capability, able to process both textual and imaging data, and was claimed to perform “exceptionally well in computer vision tasks” [57]. Multiple published dental studies have used Gemini and 2.5 Pro appeared to perform well in radiographic assessment [58]. In an earlier benchmarking test, Gemini 2.5 Pro had a better “scientific” performance compared to GPT o3 [59]. Gemini 2.5 Pro (end point identifier: gemini-2.5-pro; stable release since June 2025, no fixed-dated snapshot identifier available) was used for this study.

Claude Sonnet 4 has shown emerging potential in dental research. Claude Sonnet 4 is a “hybrid reasoning model” able to extract information from visual data and has been suggested for practical throughput [55]. A recent comparison tested improvements between the previous and updated models, and Claude was the only LLM that showed a substantial gain in diagnostic ability for oral diseases, suggesting that it has the greatest potential for further advancement [60]. In the medical field, Claude even outperformed 2 board-certified radiologists (and 9 other LLMs) in diagnostic performance in radiology cases [61]. Another study compared the latest LLMs in answering multiple-choice, text and image-based questions, and Claude was found to be the second most accurate model [62]. Specifically, Claude excelled as one of the best models in the periodontal and radiology domains, answering 87.9% and 86.7% of the questions correctly, which made it a suitable model to be included in this study. Therefore, Claude Sonnet 4 (end point identifier: claude-sonnet-4‐20250514; publicly released May 22, 2025) was used for this study.

### Prompting and Computational Approach

Each scenario, including all the input data, was followed by the same prompt (Textbox 1). The prompt asked for a periodontal risk assessment, including a status quo (ie, a provisional diagnosis) and translation into (preventive) treatment strategies. To enable prevention prior to the actual onset, a numerical periodontal risk score was used to estimate an individual’s probability of developing periodontitis in the near future (onset or progression to severe periodontitis). This risk score was based on multiple influencing factors rather than on periodontal grading, which reflects the categorical progression of periodontal destruction already accrued over preceding years and is based only on smoking and diabetes. The prompt was initially drafted based on clinical guidelines, commonly available EHR data, and expert input and refined through multiple iterations to increase clarity and relevance of the outputs. Prompt engineering took place in close consultation with a periodontist to ensure clinically appropriate outputs and with computer science researchers to optimize engineering settings. Prompting strategies for dental educators [63] were applied to guarantee that the prompt reflected the context, role, task, outputs, specificity, clarity, and audience targeting. The phrasing was carefully guided to prevent unnecessary repetition of input data, and a maximum word count of 150 was set, thereby balancing model comprehension with clinical readability. An additional scenario (scenario 0) was used to conduct pilots in all M-LLMs until the prompt reached outputs as close as desired, ensuring that scenarios 1 to 10 were never exposed to the prompt optimization process and thus reflected unbiased model responses to the finalized prompt. The input data were structured to fit the prompt format.

Textbox 1. **Prompt provided with each scenario.**

Assume that you are a periodontist, guiding dental practitioners. Use the following textual and image input data; ensure you include any plaque-retentive factors if detected on the image. Propose a provisional diagnosis and estimate the risk for developing (severe) periodontitis in the next 5 years between 0% and 100%, following the 2017 Classification of Periodontal and Peri-Implant Diseases and Conditions (American Academy of Periodontology and European Federation of Periodontology). Based on this, suggest a preventative appointment-based treatment plan that includes specific strategies and expected outcomes targeted to this case in accordance with the most recent dental and periodontal guidelines. Keep your answer between 100 and 150 words, avoid repeating my input features, but do not leave out essential details in your suggestions.

Since the settings of each model slightly differ, we aimed for consistent parameters—a concise writing style, with temperature and top-p set as low as possible (temperature set to 0 if configurable; otherwise, default developer settings were used)—to reduce randomness and creativity in the model’s outputs. The maximum token length and penalties were left at their default values. All processing was conducted on the same machine, with refreshed chat sessions for each prompt. We used the developer environments (OpenAI Playground, Google AI Studio, and Anthropic Console) rather than the standard user-facing chat interfaces (ChatGPT, Gemini, and Claude), as controlled parameter settings and reproducibility of model outputs are essential for scientific research. Claude was free to use, while costs for Gemini and GPT remained below Aus $20 (approximately US $12) for the entire study.

Three M-LLMs were applied to 10 scenarios, and 30 outputs were generated in total. Radiographs were uploaded in full dimensions as PNG files (approximately 2800×1400 pixels). The time required to assess and generate each output was also collected and estimated if not automatically generated. Each prompt was run 3 times per model and manually checked for content-level differences. As no substantive differences were found, one output per model per scenario was selected for expert rating. All outputs were collected for each model by July 22, 2025. The raw outputs generated by the M-LLMs were directly used and evaluated without additional postprocessing. The formatting was harmonized to avoid recognizable style features (eg, bold text), but structural markers and model-specific phrasing in the outputs may have survived the harmonization process, leaving a residual detection bias risk.

In addition to the AI-generated outputs, human-generated outputs were produced by a periodontist. This periodontist was asked to conduct a comprehensive periodontal risk assessment and recommend preventive strategies, based on the exact same scenarios, radiographs (in PNG), and prompt. These manually generated outputs were timed and will serve as the preliminary reference. The periodontist added 10 more outputs to the 30 AI-generated outputs, resulting in a total of 40 outputs.

### Evaluation of the Generated Responses

All generated outputs were evaluated using an online evaluation tool by 3 periodontal experts. All experts were qualified periodontists with substantial clinical experience, ensuring a high level of expertise. Prior to the evaluation, they received detailed instructions explaining the scoring system and criteria. They were informed that they were evaluating AI-generated outputs exclusively. Once data collection was closed, it was disclosed that some outputs were human-generated, thereby minimizing the risk of detection bias. Experts were asked to first review each clinical scenario individually, without seeing any generated outputs, to establish context. They then evaluated the 4 outputs for each scenario and assessed (1) diagnostic ability, (2) risk assessment ability, (3) extraction of relevant radiographic findings, (4) treatment planning ability, (5) overall completeness, and (6) specificity of the output. Ratings were assigned on a 5-point Likert scale (1=“very poor,” 2=“poor,” 3=“acceptable,” 4=“good,” 5=“very good”), with scores below 3 considered unacceptable. Expert ratings were used to evaluate multiple aspects of the outputs, including logical reasoning, perceived relevance, overtreatment or undertreatment, and overall preferences, which cannot be easily assessed in a true/false manner. The complete evaluation form can be found in Multimedia Appendix 2.

### Statistical Analyses

Analyses were conducted using IBM SPSS Statistics version 30, and significance was set at *P*<.05. Interrater agreement among all experts was calculated to determine whether their scores were consistent and reliable and could be merged for analyses (Table 1). This was performed by an intraclass correlation coefficient (ICC; 2-ways random-effects ANOVA). An overall ICC of 0.722 showed an acceptable agreement to merge their scores. The agreement about outputs generated by the periodontist (ICC=0.615) was lower than the agreement about outputs generated by AI. The ICC for Gemini as a source was 0.781, GPT 0.645, and Claude 0.622, with a combined ICC of 0.744 for all AI-generated outputs. Pairwise comparisons showed the agreement between specific experts, using the same 2-way random effects model, with Bonferroni correction applied to post hoc pairwise comparisons. Experts 1 and 2 showed good overall agreement (ICC=0.733), but both pairings with expert 3 showed lower overall agreement levels (0.579 and 0.587). The agreement between experts 3 and 1 was remarkably low concerning the periodontist’s outputs (ICC=0.318).

**Table 1.** Interrater agreements among all experts, between specific experts, and per sources (specific models, all AI models combined, and the periodontist).

Interrater agreement	Overall	Gemini 2.5 Pro	GPT o1 Pro	Claude Sonnet 4	M-LLMs^a^	Periodontist
All experts	0.722	0.781	0.645	0.622	—^b^	0.615
All experts	0.722	—	—	—	0.744	0.615
Experts 1 and 2	0.733	—	—	—	0.764	0.670
Experts 2 and 3	0.587	—	—	—	0.620	0.505
Experts 1 and 3	0.579	—	—	—	0.592	0.318

a LLM: large language model.

b Not applicable.

To analyze each M-LLM’s ability to assess the risk of developing or progressing periodontitis, the proportion of all acceptable scores (3-5) was calculated from all 720 scores (240 ratings from 3 experts), reflecting the frequency of minimally acceptable performance on the original 5-point Likert scale. An overall mean score, SD, total score, and the proportion of the maximum score were calculated for each model by using descriptive statistics to capture the magnitude of its performance (to differentiate between “acceptable” and “very good” assessments). Although Likert scale scores are ordinal in nature, simulation studies have shown that these scores can be validly analyzed as continuous variables using parametric methods without elevating error rates [64]. Parametric analyses were therefore applied in an exploratory and supplementary capacity to better capture subtle differences between models. A repeated measures ANOVA with Bonferroni-corrected post hoc pairwise comparisons was used to test whether the mean scores were significantly different from other models and the periodontist and across specific abilities (4 abilities and 2 output quality indicators) within each model. A repeated measure ANOVA also tested whether scores differed across scenarios within each model. Independent *t* tests analyzed whether scenario-specific scores depended on the presence or absence of ABL and the extent of plaque-retentive factors (no or ≤5 lesions vs >5 lesions) in the OPG. The correctness of radiographic findings mentioned in the outputs was analyzed quantitatively for alveolar bone levels using confusion matrices, comparing the radiographic findings with those of 2 oral radiologists as the reference. Any disagreements between the radiologists were resolved through calibration to reach consensus. Plaque-retentive factors were inconsistent, often described in words rather than expressed by tooth numbers, which prevented quantitative comparison; these were therefore qualitatively analyzed.

## Results

### Participants

Three periodontal experts evaluated all generated outputs regarding periodontal assessments. A relatively young periodontist, a middle-aged periodontist, and a retired periodontist were included; they had extensive clinical experience and were all active in periodontal research (Table 2).

**Table 2.** Periodontal experts who rated the generated outputs and their experience.

Experts	Graduated as a periodontist	Clinical experience (y)	Periodontal research experience (y)
Periodontal expert 1	5 years ago	13	4
Periodontal expert 2	14 years ago	24	14
Periodontal expert 3	38 years ago	38	41

### Performance

#### Acceptable Scores

The ability of the 3 M-LLMs to assess periodontal scenarios was evaluated across all individual ratings, and the proportion of acceptable (≥3), poor (2), and very poor (1) scores is shown in Figure 1. GPT, Claude, and the periodontist each achieved 86.7%, 85.6%, and 87.8% acceptable scores, respectively, while Gemini scored only 59.4% acceptable score. GPT and Claude had 12.2% and 12.8% poor scores and 1.1% and 1.7% very poor scores, respectively. The periodontist had 10.0% poor and 2.2% very poor scores, which were consistently related to radiographic misinterpretation. Notably, radiographic findings rated as unacceptable, particularly incorrect alveolar bone level assessments, had downstream effects on the diagnosis, risk, and treatment planning due to error propagation, which was observed in 78.2% of all unacceptable scores (Gemini 60/73, GPT 19/24, Claude 18/26, and periodontist 15/22). Only 4.6% of all scores represented radiographically independent errors.

**Figure 1.**
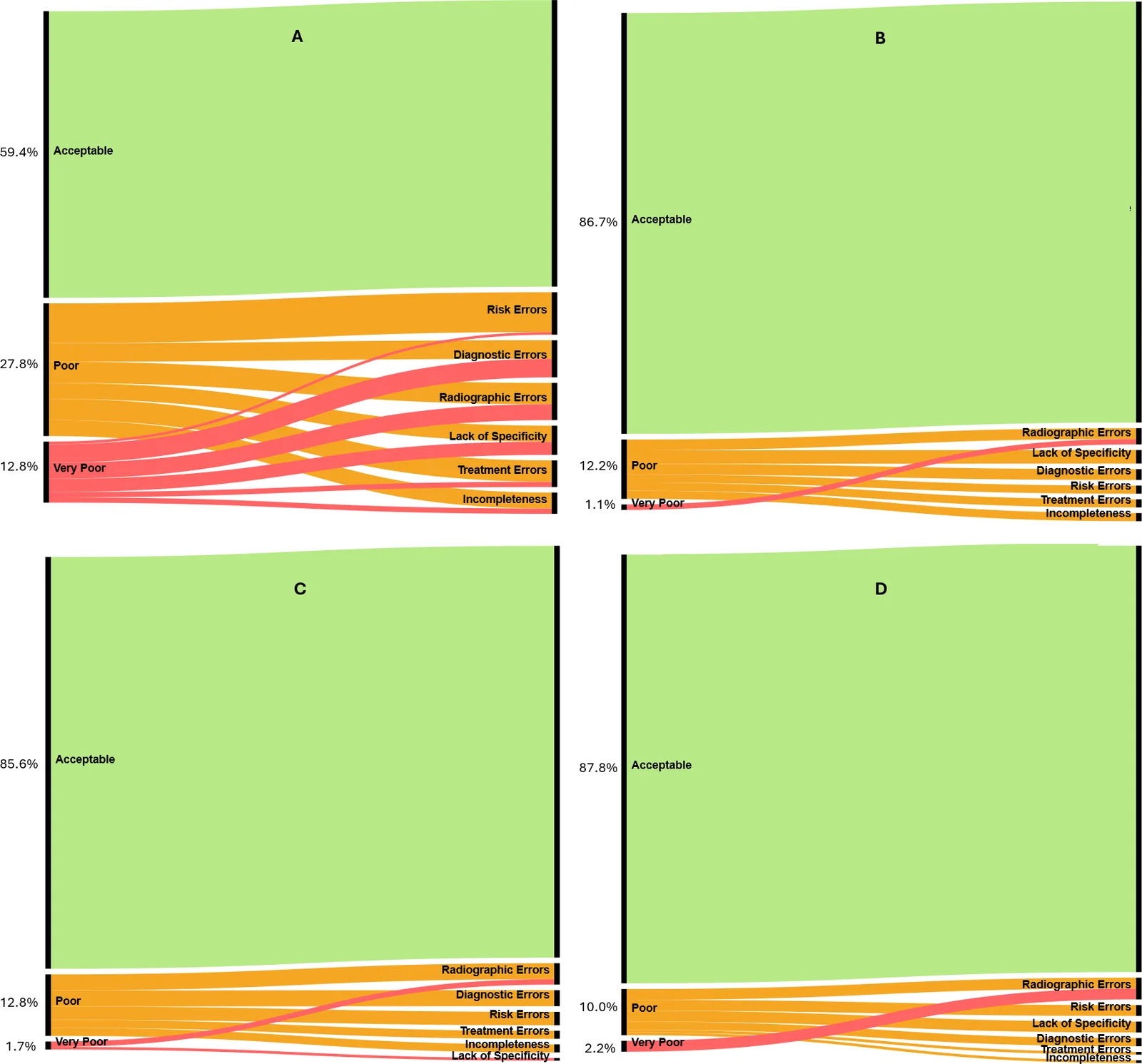
The proportion of acceptable and unacceptable scores, sorted per multimodal large language models (M-LLMs) and the periodontist. (A) Gemini 2.5 Pro; (B) GPT o1 Pro; (C) Claude Sonnet 4; and (D) periodontist.

#### Total Score

Multimedia Appendix 3 presents the overall ratings of the 3 models and the periodontist. Gemini achieved the lowest mean score of 3.07 (SD 1.33), while GPT and Claude scored slightly below 4 (mean 3.82, SD 1.03 and mean 3.77, SD 1.08, respectively), and the periodontist obtained the highest mean score of 4.01 (SD 1.10). To facilitate comparison, a total score was calculated by summing all ratings and expressing this as a proportion of the maximum possible score. Gemini performed significantly worse (61.3%) than GPT, Claude, and the periodontist (*P*≤.002). GPT and Claude reached comparable levels, scoring 76.4% and 75.3%, respectively, while the periodontist achieved 80.1%. The difference between GPT and the periodontist was not significant (*P*=.31), whereas Claude scored significantly lower than the periodontist (*P*=.046). In terms of efficiency, Gemini assessed and generated outputs in an average of 37 (SD 5) seconds, GPT required 3 minutes and 22 seconds (SD 54 s), and the periodontist required 5 minutes and 57 seconds (SD 1 min and 45 s). Although exact timings were not available, Claude read and produced outputs even faster than Gemini, with a consistent, estimated time of approximately 10 seconds.

Scores varied significantly between experts (*P*<.05). Expert 3 systematically assigned significantly lower scores than experts 1 and 2 (*P*<.05). For the outputs generated by the periodontist, opposing scoring patterns among experts were observed, as reflected by negative ICCs. In contrast, the ICC for scenarios 9 and 10 was relatively high (0.804 and 0.750, respectively), while having the lowest absolute scores. Generally, the scores also varied significantly across scenarios (*P*<.001). Overall, higher scores were observed in scenarios 1, 6, 7, and 8. Scenarios with normal alveolar bone levels scored significantly higher than those with ABL (*P*<.001), and scenarios with minimal plaque-retentive factors received higher scores than those with multiple plaque-retentive factors (*P*<.05). In these complex scenarios, GPT, Claude, and the periodontist still maintained acceptable scores, whereas Gemini fell below the acceptable threshold in scenarios with ABL and with multiple plaque-retentive factors on the OPG. The time of assessment was also influenced by scenario complexity. For the periodontist and GPT, the generation times increased with scenario complexity (eg, scenarios 2, 3, 8, 9, and 10), whereas Gemini maintained relatively consistent completion times across all scenarios (Gemini: 31‐47 s; GPT: 1 min 51 s–4 min 26 s; Claude: approximately 10 s; periodontist: 3‐8 min 30 s; Multimedia Appendix 4).

#### Abilities and Quality of Outputs

Figure 2 shows the mean scores for specific abilities in periodontal assessment and quality criteria of outputs (maximum composite score=30). Across the 3 models and the periodontist, radiographic interpretation consistently received lower scores than other abilities, with Gemini scoring below 3 (translated as unacceptable). GPT and Claude generally scored slightly below 4 across most abilities, whereas the periodontist consistently scored slightly above 4 (translated as good). Among the AI-generated outputs, diagnostic ability and completeness of outputs were rated highest. Gemini scored significantly higher on completeness than the specificity of outputs (*P*=.01). GPT’s ability to analyze radiographs was significantly lower than its ability to diagnose (*P*=.005) and assess the risk (*P*=.009). For Claude and the periodontist, performance did not vary significantly across specific abilities or output qualities.

**Figure 2.**
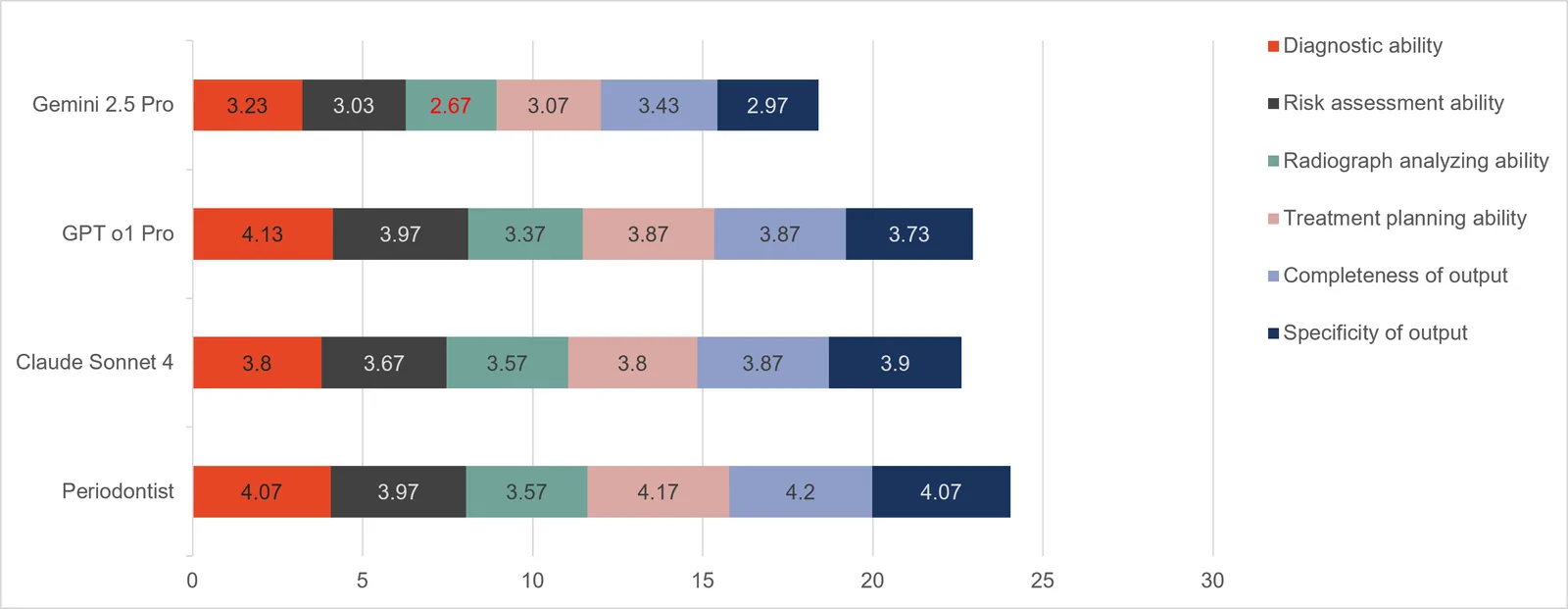
The mean scores for specific abilities in periodontal assessment and quality criteria of outputs.

Zooming in on the periodontal risk assessments, Figure 3 shows the risk estimates by 3 LLMs and the periodontist across 10 clinical scenarios. The mean absolute error (MAE) reflects the average deviation from the periodontist, with GPT o1 Pro showing the closest alignment (MAE=9.0%), followed by Claude Sonnet 4 (MAE=22.5%), while Gemini 2.5 Pro deviated most (MAE=33.5%), systematically overestimating the periodontal risk. Unacceptable risk assessments were predominantly observed for Gemini 2.5 Pro, though scenario 10 also yielded an unacceptable rating by the periodontist. Although missing data were present in 6 scenarios, no M-LLM flagged or warned about the absence of data. While the periodontist noted this omission in 3 of these scenarios, only one hindered the risk assessment, with the resulting estimate nonetheless rated as acceptable.

**Figure 3.**
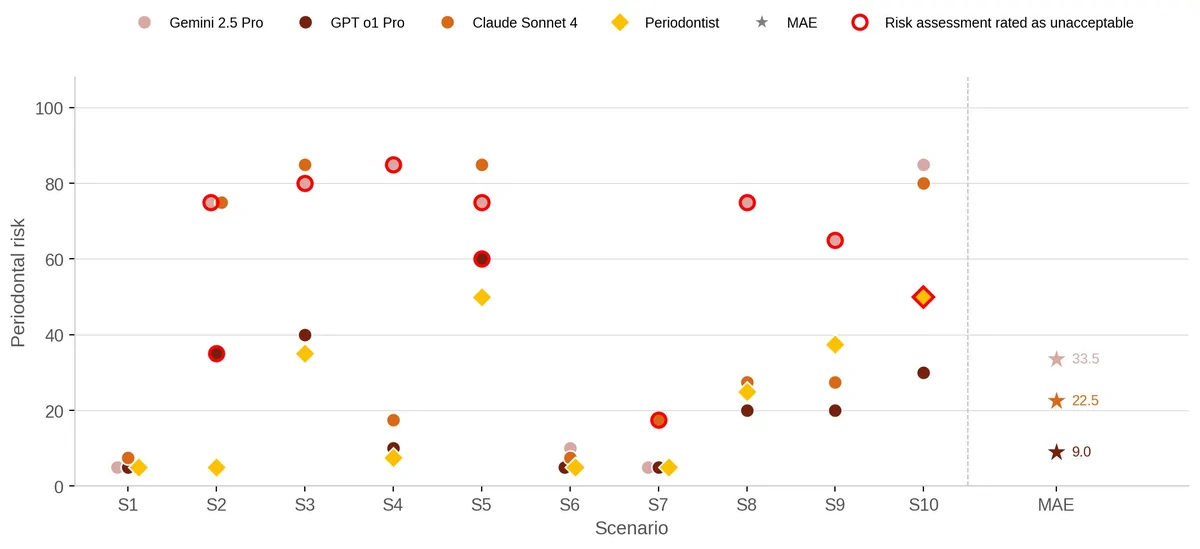
The periodontal risks estimated by the multimodal large language models (M-LLMs) compared to those of the periodontist. MAE: mean absolute error.

#### Ability to Analyze Radiographs

Given that radiographic analysis received the lowest, often unacceptable ratings, we examined specific radiographic findings reported in the outputs. The confusion matrices in Figure 4 show that ABL was present in 4 scenarios and provide insight into how often the models correctly included or excluded ABL in their outputs, ideally reflected by a diagonal pattern. Although the prompt did not explicitly request to include the bone level in the output (only instructing to “analyze the panoramic radiograph attached”), Gemini and the periodontist included bone-level evaluations in only 5 and 4 of the outputs, respectively (not limited to cases with ABL), whereas GPT and Claude assessed bone levels in nearly all outputs. When reported, 8/10 and 7/9 bone levels were correctly identified, respectively. Gemini included partially erupted third molars as the dominant plaque-retentive factor in 9/10 outputs, and the periodontist in 7/10 outputs, whereas GPT and Claude included this factor only twice and once, respectively. GPT and Claude primarily reported calculus (in 9 and 10 outputs, respectively, while only 5 OPGs had actual calculus) and restorative margin defects (in 7 and 8 outputs, respectively, while only 1 OPG showed an actual margin defect) as detected plaque-retentive factors. The location of these factors was often nonspecific, for example, “lower anterior teeth” or “generalized” for calculus, and “posterior region” or first molars for restorative margin defects. Outputs referred to the commonly expected sites for each pathology rather than image-based findings. Interdental carious defects were reported in 2 outputs by Claude and in 6 outputs by the periodontist, but were included in a parallel treatment plan rather than linked to periodontal risk. According to oral radiologists, interdental carious defects were present in 8 OPGs, but they were rarely included as a periodontal risk factor in any of the outputs. The absence of plaque-retentive factors in the outputs does not necessarily indicate the absence of detection; however, the findings in the output were considered relevant for assessing the periodontal risk. An example of the outputs can be found in Multimedia Appendix 5.

**Figure 4.**
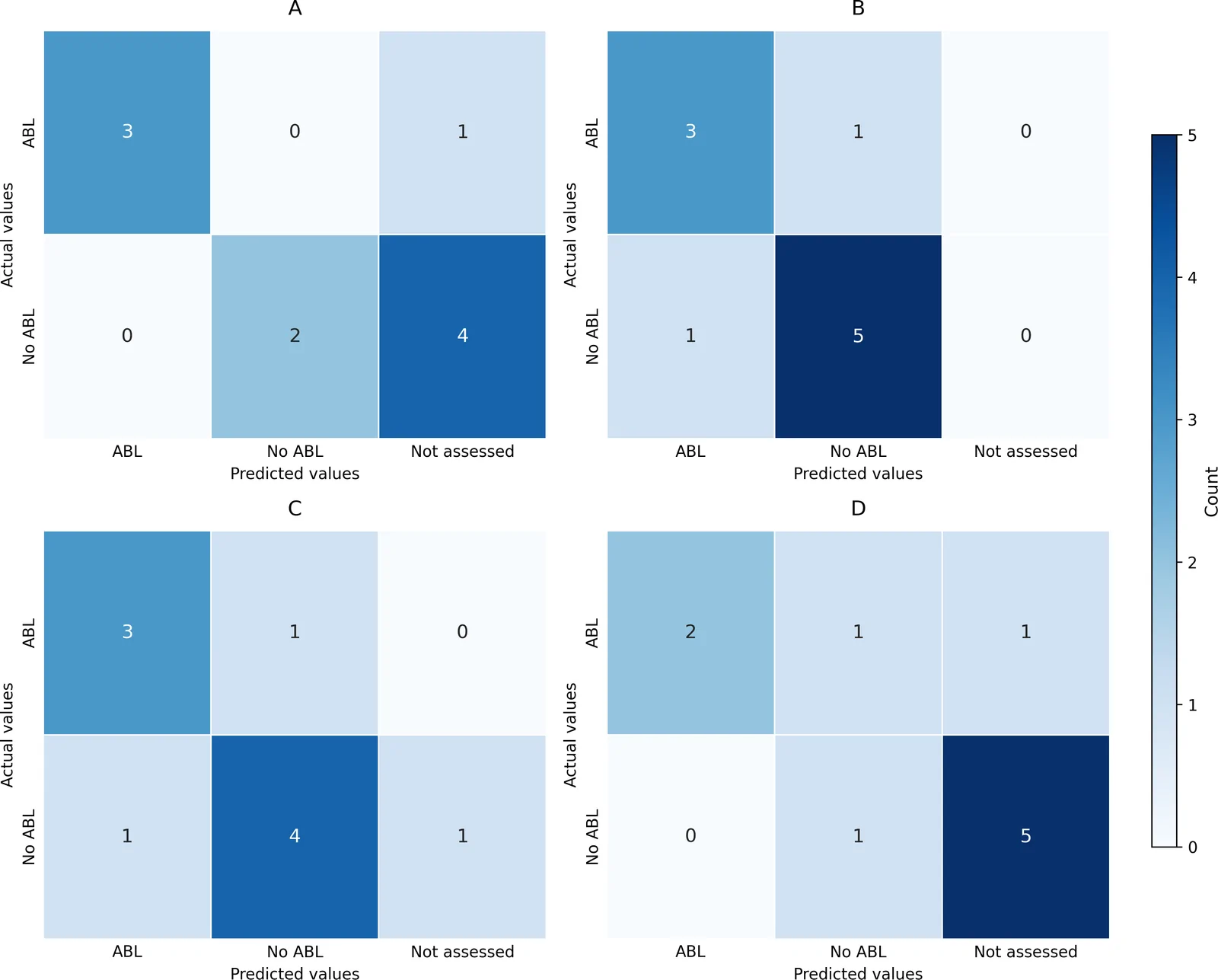
Confusion matrices of bone levels included in the outputs, compared with the “actual” bone levels as determined by oral radiologists, shown for each model and periodontist. (A) Gemini; (B) GPT; (C) Claude; and (D) periodontist. ABL: alveolar bone loss.

## Discussion

### Principal Findings

This study evaluated the ability of M-LLMs to assess periodontal scenarios based on textual and imaging input data. GPT o1 Pro’s and Claude Sonnet 4’s ability to diagnose, assess the risk, extract radiographic findings, suggest treatment, and the completeness and specificity of the outputs were generally good, with 85.6% and 86.7% acceptable scores and risk estimations close to those of the periodontist. Gemini performed acceptably in 59.4% of cases, specifically scoring unacceptably in its extraction of radiographic findings. This lower performance on image-based questions is consistent with a recent study [62], but generally, the literature about the best M-LLM to analyze oral radiographs varies enormously [65]. GPT and Claude performed comparably to the periodontist, and Gemini was evaluated as significantly less satisfactory than the periodontist and other models. LLMs performed worse in complex scenarios but were still at an acceptable level. It is important to note that, based on the opinion of periodontal experts, the periodontist achieved 80.1% of the maximum score, indicating that human-generated outputs are not perfect either. However, the evaluated “imperfections” in the generated periodontal outputs may reflect actual limitations of the model, the ambiguous “truth” perceived by the experts rating the outputs, or a lack of specificity in prompts.

### Limitations

The main limitation of this study is that the reference standard was provided by a single periodontist, limiting its reliability and should therefore be considered preliminary rather than a definitive clinical standard. Despite efforts to recruit additional periodontists, no second provider could be found. This limitation is particularly concerning, given that raters showed lower agreement on human-generated outputs than on AI-generated outputs (ICC: 0.615 vs 0.744). The low interrater agreement between experts 1 and 3 even reflected that the periodontist’s outputs were valued contradicting—ideal by one, yet far from it by the other. One expert consistently assigned lower ratings across all outputs, including those of the periodontist. This could reflect expectancy bias of a single rater, assuming only this rater believed all outputs were AI-generated, given the blinded design. However, the low agreement is more appropriately attributed to expert 3’s consistent, diverging scoring based on the most extensive periodontal experience and expertise among the 3 raters, reflecting a higher clinical standard. This divergent pattern has lowered the scores but reflects clinical variance rather than a methodological flaw and was therefore not considered grounds for excluding this expert’s data. The low ICC is therefore a limitation of the small sample rather than a reflection of invalid scoring. Calibration to reach consensus could have been applied to increase the reliability of the reference, but simultaneously, this limitation reflects the subjectivity in periodontal risk assessments, the absence of a universally accepted ideal outcome, and the need for a more robust reference standard in future research.

A second limitation relates to the ability of M-LLMs to assess radiographs. The prompt lacked specificity, as it did not explicitly define which radiographic factors should be evaluated, such as alveolar bone levels. Similarly, it was impossible to determine whether the absence of plaque-retentive factors in the output reflected true absence, nondetection in the image or model assumptions about the relevance for periodontal risk and prevention. Despite extensive prompt exploration during the pilot stage, no prompt consistently yielded valid radiographic outputs, with any localization (tooth-level, sextant, and upper/lower or left/right) remaining unsuccessful. Word limits may also have affected the quality of reporting. While radiographic performance issues were not solely driven by output length (as 70% of radiographic errors were rated as complete), word limits might have forced prioritizing of certain clinical data over others. Similarly, preventing verbatim repetition of the input data might also have suppressed the flagging of essential missing data. This likely reflects a broader ceiling in current M-LLM’s radiographic interpretation rather than an issue solely of prompt design. A related consideration is that these M-LLM APIs may preprocess high-resolution images before analysis (eg, through resizing, compression, or tiling), but the exact procedures are generally not disclosed by model providers. Consequently, part of the observed performance may reflect information loss introduced during image processing rather than the models’ ability to assess radiographs alone. The resulting incorrect radiographic findings may have caused error propagation, affecting not only AI-generated outputs but also clinical reasoning by the periodontist. Particularly incorrect alveolar bone levels directly led to incorrect diagnoses, risks, and treatment recommendations. Models might have prioritized their own radiographic interpretation over the available clinical data by generating a diagnosis, reflecting good but misdirected medical reasoning rather than purely computer vision failures. Therefore, the present findings should be interpreted as an evaluation of real-world system outputs rather than a direct assessment of the models’ underlying vision capabilities.

Third, the generalizability of the findings is subject to external and internal concerns. The external generalizability is limited by the use of only 10 clinical vignettes. These vignettes were constructed by combining publicly available OPGs, which may have been included in the models’ training data, with synthetic EHR variables, potentially affecting the authenticity and representativeness of the cases. Future studies should include a larger and more diverse set of real-world clinical scenarios. Internally, content-level consistency across 3 iterations per prompt was manually verified and subjectively confirmed to be high. However, only one of these 3 outputs was ultimately presented to the raters. Presenting all 3 blinded would have provided a higher degree of internal generalizability and a more robust estimate of the M-LLM’s mean performance and error rates.

### Practical Implications and Future Research

This study shows that nowadays M-LLMs are well-suited for textual tasks. They were benchmarked against a periodontist as a reference, rather than aiming for perfection. Given that these models are already widely used by patients and clinicians [56,66], it is crucial to understand their capabilities, limitations, and potential risks. Waiting for models to achieve 100% accuracy is neither realistic nor aligned with how society is already adopting them. Instead, current implementations must be guided, where taking into account the implications is of the utmost importance.

Although not directly investigated in this study, we expect that such models may hold potential for summarizing EHRs in referrals, providing patient feedback through online portals, and supporting public health screenings, given their strong medical reasoning and applied knowledge. However, their outputs remain conceptual rather than finalized and require human review before clinical use. The current models should not be used for specific vision tasks yet (such as caries detection), especially not at the tooth level, as they seem incapable of numbering teeth, sextants, or any other input-based location. Specific vision models remain necessary, although newer M-LLMs, such as GPT-5, are expected to improve in this area. In this study, some outputs were still unacceptable and potentially harmful without accurate radiographic extraction, so multimodal use should currently be limited to general image detection tasks. EHR software companies could consider implementing LLMs based on textual input, but they should require a minimum number of input variables to conduct the assessment. Due to the risk of inaccurate results stemming from insufficient input data, tasks should be conducted carefully to avoid garbage-in-garbage-out consequences. Future research should start with trials testing digitally integrated LLMs without immediately disclosing outputs to clinicians, allowing summaries and referrals to be evaluated and models to be further optimized. Direct patient use is discouraged due to the need for precise prompts, complete input data, and translated reporting suitable for patients.

### Conclusions

M-LLMs show promising abilities in their periodontal risk assessment, with Claude and particularly GPT performing comparably to the periodontist, receiving acceptable scores in 85.6% and 87.8% of cases. Gemini performed significantly worse, with only 59.4% acceptable scores and consistently overestimating the periodontal risk. Across all models, unacceptable elements consistently were related to errors in radiographic interpretation, though refined prompting or newer model versions may improve this. Notably, even when radiographic findings were incorrect and plaque-retentive factors were absent, outputs were still rated well, indicating that EHR data alone provide a substantial basis. For clinical applicability, M-LLMs must at least perform comparably to a periodontist and meet the quality standards set by periodontal experts—a bar that GPT and Claude appear to approach.

## Supplementary material

10.2196/87781 Multimedia Appendix 1 Vignettes, describing periodontal scenarios.

10.2196/87781 Multimedia Appendix 2 Output evaluation form.

10.2196/87781 Multimedia Appendix 3 Overall scores of each model and the periodontist and the time of conduction. (A) Gemini 2.5 Pro; (B) GPT o1 Pro; (C) Claude Sonnet 4; (D) periodontist.

10.2196/87781 Multimedia Appendix 4 Variation in score and generation time per scenario, sorted per M-LLM/periodontitis.

10.2196/87781 Multimedia Appendix 5 Example of outputs per model and the periodontist.

## Abbreviations

ABL: alveolar bone loss
EHR: electronic health record
ICC: intraclass correlation coefficient
*ICD*: *International Classification of Diseases*
LLM: large language model
M-LLM: multimodal large language model
MAE: mean absolute error
OPG: orthopantomogram
PSI: Periodontal Screening and Recording Index
TRIPOD: Transparent Reporting of a Multivariable Prediction Model for Individual Prognosis or Diagnosis
