# Supplementary material for: Periodontitis Risk Assessment and Prevention Planning: Comparative Study of Multimodal Large Language Models and Periodontist Evaluations
Source: J Med Internet Res. 2026 Oct 2;28:e87781. doi: 10.2196/87781 (PMC13632909; doi:10.2196/87781)
Supplement: Supplementary file 1 [file jmir-v28-e87781-s002.pdf]

Assess each generated response by its:	5= very good	4= good	3= accep table	2= poor	1= very poor
**Ability to diagnose**<br>*Consider the correctness of the provisional diagnosis and alignment with the latest guidelines.*					
**Ability to assess the risk**<br>*Consider the correctness of the estimated risk, logic made regarding accountable risk factors and causes.*					
**Ability to extract radiographic findings**<br>*Consider the correctness and completeness of (solely relevant) radiographic findings included in the response.*					
**Ability to suggest treatment**<br>*Consider the correctness of (preventive) treatment suggestions, alignment with the latest guidelines, and potential under- or overtreatment.*					
**Overall completeness of the response**<br>*Consider the presence or missing of all required information.*					
**Overall specificity of the response**<br>*Consider the level of detail, scenario-specificity, and applicability of the response (versus a too general response).*					
