# Supplementary material for: Periodontitis Risk Assessment and Prevention Planning: Comparative Study of Multimodal Large Language Models and Periodontist Evaluations
Source: J Med Internet Res. 2026 Oct 2;28:e87781. doi: 10.2196/87781 (PMC13632909; doi:10.2196/87781)
Supplement: Supplementary file 2 [file jmir-v28-e87781-s005.pdf]

Gemini 2.5 pro

Based on the provided data, the provisional diagnosis is Generalized Periodontitis, Stage III, Grade C.

The radiograph shows significant vertical bone loss, particularly on the mesial aspects of the mandibular first molars, inconsistent with the patient's young age. Plaque-retentive factors include partially erupted and impacted third molars (38, 48) and potential crowding. Given the rapid progression (Grade C), the risk of further severe periodontitis within 5 years is high (~85%).

A comprehensive treatment plan should follow the EFP S3 guidelines:

1. Step 1: Intensive, personalized oral hygiene instruction focusing on effective brushing (2x/day) and daily interdental cleaning with appropriately sized interdental brushes.
2. Step 2: Full-mouth non-surgical debridement. Consider extraction of the impacted third molars to eliminate plaque traps.
3. Step 3: Re-evaluate after 8-12 weeks. The expected outcome is BOP <10% and probing depth reduction. Persistent deep vertical defects may require surgical regenerative therapy.
4. Step 4: Lifelong supportive periodontal care every 3 months is crucial to maintain stability.

GPT o1 pro

Radiographic assessment shows no major alveolar bone loss or extensive restorations, though small amounts of calculus are likely around posterior sites, creating plaque-retentive niches.

Clinically, mild inflammatory changes with occasional bleeding but without advanced pocketing suggest an early periodontal condition consistent with plaque-induced gingivitis under the 2017 classification.

The estimated risk of developing severe periodontitis within five years is approximately 10%.

A focused prevention plan is recommended: enhanced homecare instruction with more frequent brushing, regular interdental cleaning, and professional scaling at six-month intervals. Particular attention should be paid to thorough cleaning of posterior areas prone to plaque buildup. Employing additional measures, such as topical fluoride and tailored dietary guidance, will help limit caries risk. Expected outcomes include reduced bleeding, improved hygiene, and stable periodontal support, minimizing progression toward more serious disease.

Claude Sonnet 4

Looking at this panoramic radiograph of a 20-year-old female patient, I observe:

Radiographic findings: Generally healthy bone levels with no significant horizontal or vertical bone loss. Some scattered calculus deposits visible, particularly in posterior regions. No obvious caries or defective restorations detected.

Provisional diagnosis: Gingivitis (Stage I, Grade A periodontitis risk)

5-year severe periodontitis risk: 15-20% Risk factors: suboptimal oral hygiene (once daily brushing, irregular interdental cleaning), visible calculus deposits, 20% bleeding on probing. Protective factors: young age, non-smoker, systemically healthy.

Treatment plan:

- Professional scaling/debridement to remove calculus
- Oral hygiene instruction emphasizing twice-daily brushing and daily interdental cleaning
- 3-month recall appointments initially, extending to 6 months once gingivitis resolves
- Monitor plaque scores and bleeding indices
- Expected outcome: Resolution of gingivitis within 2-3 months with proper home care compliance

Early intervention at this stage should prevent progression to periodontitis given her young age and absence of systemic risk factors.

Periodontist

Provisional Diagnosis: Based on the X-rays, we can detect no marginal bone loss. Clinically, the patient has a CPI score of 2, with 20% BoP and the presence of subgingival calculus

Her risk for developing (severe) periodontitis in the next 5 years is closer to 5-10%.

Preventive appointment-based treatment plan: An appointment at a general dental practice for treatment of the caries is needed. A motivation of her oral hygiene habits is also highly recommended (electric, 2 times per day with interdental brushing), as well as advise on eating habits (eating moments and sugar consumption) to help mitigate the progression of interdental caries. Afterwards, a dental cleaning is advisable to remove the calculus the patient currently has, followed by a follow-up appointment(s) to evaluate the compliance/progress of the patient’s periodontal health and the removal of teeth 28 and 38, which are impacted and could cause caries and periodontal issues.
