# Supplementary material for: Periodontitis Risk Assessment and Prevention Planning: Comparative Study of Multimodal Large Language Models and Periodontist Evaluations
Source: J Med Internet Res. 2026 Oct 2;28:e87781. doi: 10.2196/87781 (PMC13632909; doi:10.2196/87781)
Supplement: Supplementary file 3 [file jmir-v28-e87781-s001.docx]

Scenario 1:

Input*:*

*Assume that you are a periodontist, guiding dental practitioners. Use the following textual and image input data; ensure you include any plaque-retentive factors if detected on the image. Propose a provisional diagnosis and estimate the risk for developing (severe) periodontitis in the next 5 years between 0-100%, following the 2017 Classification of Periodontal and Peri-Implant Diseases and Conditions (AAP and EFP). Based on this, suggest a preventive appointment-based treatment plan that includes specific strategies and expected outcomes targeted to this case in accordance with the most recent dental and periodontal guidelines. Keep your answer between 100-150 words, avoid repeating my input features, but do not leave out essential details in your suggestions.*

*Image: analyze the panoramic radiograph as attached*

*Clinical findings:*

- *periodontal screening index (CPI): 0*
- *bleeding on probing score: missing*
- *plaque score: missing*

*Health behavior:*

- *smoking: no*
- *oral hygiene: brushes electric, 2 times per day for 2 minutes*
- *interdental cleaning: uses dental sticks 5 times per week*

*Patient data:*

- *sex: woman*
- *age: 16 years old*
- *medical conditions: no*

(add radiograph: Train_74)

Scenario 2

Input*:*

*Assume that you are a periodontist, guiding dental practitioners. Use the following textual and image input data; ensure you include any plaque-retentive factors if detected on the image. Propose a provisional diagnosis and estimate the risk for developing (severe) periodontitis in the next 5 years between 0-100%, following the 2017 Classification of Periodontal and Peri-Implant Diseases and Conditions (AAP and EFP). Based on this, suggest a preventive appointment-based treatment plan that includes specific strategies and expected outcomes targeted to this case in accordance with the most recent dental and periodontal guidelines. Keep your answer between 100-150 words, avoid repeating my input features, but do not leave out essential details in your suggestions.*

*Image: analyze the panoramic radiograph as attached*

*Clinical findings:*

- *periodontal screening index (CPI): 0*
- *bleeding on probing score: 5%*
- *plaque score: 45%*

*Health behavior:*

- *smoking: yes*
- *oral hygiene: brushes manually, 2 times per day for 1 minute*
- *interdental cleaning: 1 time per week*

*Patient data:*

- *sex: man*
- *age: 60*
- *medical conditions: Sjogren syndrome and cardiovascular disease*

(add radiograph:Train_161)

Scenario 3

Input:

*Assume that you are a periodontist, guiding dental practitioners. Use the following textual and image input data; ensure you include any plaque-retentive factors if detected on the image. Propose a provisional diagnosis and estimate the risk for developing (severe) periodontitis in the next 5 years between 0-100%, following the 2017 Classification of Periodontal and Peri-Implant Diseases and Conditions (AAP and EFP). Based on this, suggest a preventive appointment-based treatment plan that includes specific strategies and expected outcomes targeted to this case in accordance with the most recent dental and periodontal guidelines. Keep your answer between 100-150 words, avoid repeating my input features, but do not leave out essential details in your suggestions.*

*Image: analyze the panoramic radiograph as attached*

*Clinical findings:*

- *periodontal screening index (CPI): 1*
- *bleeding on probing score: 60%*
- *plaque score: missing*

*Health behavior:*

- *smoking: missing*
- *oral hygiene: brushes manually, 1 time per day for 2 minutes*
- *interdental cleaning: no*

*Patient data:*

- *sex: man*
- *age: 18*
- *medical conditions: HIV*
- *health status: obesity*

(add radiograph: Train_159)

Scenario 4:

Input:

*Assume that you are a periodontist, guiding dental practitioners. Use the following textual and image input data; ensure you include any plaque-retentive factors if detected on the image. Propose a provisional diagnosis and estimate the risk for developing (severe) periodontitis in the next 5 years between 0-100%, following the 2017 Classification of Periodontal and Peri-Implant Diseases and Conditions (AAP and EFP). Based on this, suggest a preventive appointment-based treatment plan that includes specific strategies and expected outcomes targeted to this case in accordance with the most recent dental and periodontal guidelines. Keep your answer between 100-150 words, avoid repeating my input features, but do not leave out essential details in your suggestions.*

*Image: analyze the panoramic radiograph as attached*

*Clinical findings:*

- *periodontal screening index (CPI): 2*
- *bleeding on probing score: 20%*
- *plaque score: missing*

*Health behavior:*

- *smoking: no*
- *oral hygiene: brushes manually, 1 time per day for 2 minutes*
- *interdental cleaning: not regularly*

*Patient data:*

- *sex: woman*
- *age: 20 years old*
- *medical conditions: no*

(add radiograph: Train_118)

Scenario 5:

Input:

*Assume that you are a periodontist, guiding dental practitioners. Use the following textual and image input data; ensure you include any plaque-retentive factors if detected on the image. Propose a provisional diagnosis and estimate the risk for developing (severe) periodontitis in the next 5 years between 0-100%, following the 2017 Classification of Periodontal and Peri-Implant Diseases and Conditions (AAP and EFP). Based on this, suggest a preventive appointment-based treatment plan that includes specific strategies and expected outcomes targeted to this case in accordance with the most recent dental and periodontal guidelines. Keep your answer between 100-150 words, avoid repeating my input features, but do not leave out essential details in your suggestions.*

*Image: analyze the panoramic radiograph as attached*

*Clinical findings:*

- *periodontal screening index (CPI): 2*
- *bleeding on probing score: 30%*
- *plaque score: 50%*

*Health behavior:*

- *smoking: yes*
- *oral hygiene: missing*
- *interdental cleaning: no*

*Patient data:*

- *sex: woman*
- *age: 38*
- *medical conditions: multiple sclerosis, diabetes*

*(add radiograph: Train_171)*

Scenario 6:

Input:

*Assume that you are a periodontist, guiding dental practitioners. Use the following textual and image input data; ensure you include any plaque-retentive factors if detected on the image. Propose a provisional diagnosis and estimate the risk for developing (severe) periodontitis in the next 5 years between 0-100%, following the 2017 Classification of Periodontal and Peri-Implant Diseases and Conditions (AAP and EFP). Based on this, suggest a preventive appointment-based treatment plan that includes specific strategies and expected outcomes targeted to this case in accordance with the most recent dental and periodontal guidelines. Keep your answer between 100-150 words, avoid repeating my input features, but do not leave out essential details in your suggestions.*

*Image: analyze the panoramic radiograph as attached*

*Clinical findings:*

- *periodontal screening index (CPI): 0*
- *bleeding on probing score: 2%*
- *plaque score: missing*

*Health behavior:*

- *smoking: no*
- *oral hygiene: brushes electric 2 times per day 3 minutes*
- *interdental cleaning: uses dental floss daily*

*Patient data:*

- *sex: woman*
- *age: 40*
- *medical conditions: no*

(add radiograph: Train_1443)

Scenario 7:

Input:

*Assume that you are a periodontist, guiding dental practitioners. Use the following textual and image input data; ensure you include any plaque-retentive factors if detected on the image. Propose a provisional diagnosis and estimate the risk for developing (severe) periodontitis in the next 5 years between 0-100%, following the 2017 Classification of Periodontal and Peri-Implant Diseases and Conditions (AAP and EFP). Based on this, suggest a preventive appointment-based treatment plan that includes specific strategies and expected outcomes targeted to this case in accordance with the most recent dental and periodontal guidelines. Keep your answer between 100-150 words, avoid repeating my input features, but do not leave out essential details in your suggestions.*

*Image: analyze the panoramic radiograph as attached*

*Clinical findings:*

- *periodontal screening index (CPI): 1*

*Health behavior:*

- *smoking: no*
- *oral hygiene: brushes electric, 2 times per day 3 minutes*
- *interdental cleaning: uses dental sticks regularly*

*Patient data:*

- *sex: woman*
- *age: 19*
- *medical conditions: healthy but pregnant*

(add radiograph: Train_20)

Scenario 8:

Input:

*Assume that you are a periodontist, guiding dental practitioners. Use the following textual and image input data; ensure you include any plaque-retentive factors if detected on the image. Propose a provisional diagnosis and estimate the risk for developing (severe) periodontitis in the next 5 years between 0-100%, following the 2017 Classification of Periodontal and Peri-Implant Diseases and Conditions (AAP and EFP). Based on this, suggest a preventive appointment-based treatment plan that includes specific strategies and expected outcomes targeted to this case in accordance with the most recent dental and periodontal guidelines. Keep your answer between 100-150 words, avoid repeating my input features, but do not leave out essential details in your suggestions.*

*Image: analyze the panoramic radiograph as attached*

*Clinical findings:*

- *periodontal screening index (CPI): 0*
- *bleeding on probing score: missing*
- *plaque score: missing*

*Health behavior:*

- *smoking: no*
- *oral hygiene: brushes electric 3 times a day for 4 minutes*
- *interdental cleaning: uses interdental brushes daily*

*Patient data:*

- *sex: woman*
- *age: 30*
- *medical conditions: diabetes type 2 (controlled), immunodeficiencies*

(add radiograph: Train_1451)

Scenario 9:

Input:

*Assume that you are a periodontist, guiding dental practitioners. Use the following textual and image input data; ensure you include any plaque-retentive factors if detected on the image. Propose a provisional diagnosis and estimate the risk for developing (severe) periodontitis in the next 5 years between 0-100%, following the 2017 Classification of Periodontal and Peri-Implant Diseases and Conditions (AAP and EFP). Based on this, suggest a preventive appointment-based treatment plan that includes specific strategies and expected outcomes targeted to this case in accordance with the most recent dental and periodontal guidelines. Keep your answer between 100-150 words, avoid repeating my input features, but do not leave out essential details in your suggestions.*

*Image: analyze the panoramic radiograph as attached*

*Clinical findings:*

- *periodontal screening index (CPI): 3*
- *bleeding on probing score: 5%*
- *plaque score: 7%*

*Health behavior:*

- *smoking: stopped*
- *oral hygiene: brushes electric, 3 times per day for 2 minutes*
- *interdental cleaning: uses interdental brushes daily*

*Patient data:*

- *sex: man*
- *age: 40*
- *medical conditions: rheumatoid arthritis, multiple sclerosis, hayfever allergy*

(add radiograph: Train_50)

Scenario 10:

Input:

*Assume that you are a periodontist, guiding dental practitioners. Use the following textual and image input data; ensure you include any plaque-retentive factors if detected on the image. Propose a provisional diagnosis and estimate the risk for developing (severe) periodontitis in the next 5 years between 0-100%, following the 2017 Classification of Periodontal and Peri-Implant Diseases and Conditions (AAP and EFP). Based on this, suggest a preventive appointment-based treatment plan that includes specific strategies and expected outcomes targeted to this case in accordance with the most recent dental and periodontal guidelines. Keep your answer between 100-150 words, avoid repeating my input features, but do not leave out essential details in your suggestions.*

*Image: analyze the panoramic radiograph as attached*

*Clinical findings:*

- *periodontal screening index: 4*
- *bleeding on probing score: 40%*
- *plaque score: 30%*

*Health behavior:*

- *smoking: no*
- *oral hygiene: brushes manually, 2 times per day one minute*
- *interdental cleaning: dental floss once a week*
- *Last dental visit 5 years ago*

*Patient data:*

- *sex: woman*
- *age: 50*
- *medical conditions: no*

(add radiograph: val_24)
