# Supplementary material for: Periodontitis Risk Assessment and Prevention Planning: Comparative Study of Multimodal Large Language Models and Periodontist Evaluations
Source: J Med Internet Res. 2026 Oct 2;28:e87781. doi: 10.2196/87781 (PMC13632909; doi:10.2196/87781)
Supplement: Supplementary file 4 [file jmir-v28-e87781-s004.docx]

|  | **Gemini 2.5 Pro** | **GPT o1 pro** | **Claude Sonnet 4** | **Periodontist** |
| --- | --- | --- | --- | --- |
| Scenario 1  Score  Time of generation | 4.11  30.5 sec | 4.44  3m07sec | 4.78  Approx. 10sec | 4.33  3m |
| Scenario 2  Score  Time of generation | 2.5  32.8 sec | 3.11  3m27sec | 3.33  Approx. 10sec | 4.11  6m30sec |
| Scenario 3  Score  Time of generation | 1.83  42.5 sec | 3.72  4m01sec | 4.17  Approx. 10sec | 4.33  7m |
| Scenario 4  Score  Time of generation | 1.83  39.5 sec | 4.11  3m50sec | 3.61  Approx. 10sec | 4.67  4m30sec |
| Scenario 5  Score  Time of generation | 2.78  36.0 sec | 3.11  1m51sec | 3.39  Approx. 10sec | 3.67  5m30sec |
| Scenario 6  Score  Time of generation | 3.89  47.2 sec | 4.56  2m00sec | 4.44  Approx. 10sec | 4.50  4m |
| Scenario 7  Score  Time of generation | 4.28  33.5 sec | 4.61  2m51sec | 3.50  Approx. 10sec | 4.83  5m30sec |
| Scenario 8  Score  Time of generation | 3.72  37.0 sec | 3.94  4m14sec | 4.11  Approx. 10sec | 3.83  7m30sec |
| Scenario 9  Score  Time of generation | 2.06  38.8 sec | 2.83  3m50sec | 2.56  Approx. 10sec | 2.67  8m30sec |
| Scenario 10  Score  Time of generation | 3.67  32.8 sec | 3.78  4m26sec | 3.78  Approx. 10sec | 3.11  7m30sec |
| **Significantly different scores per scenario** | p<0.001 | p<0.001 | p<0.001 | p<0.001 |
| **Scenarios with normal alveolar bone levels versus ABL** | 3.4  versus  2.5 (p<0.001) | 4.1  versus  3.5  (p<0.001) | 4.1  versus  3.3  (p<0.001) | 4.3  versus  3.6  (p<0.001) |
| **Scenarios with ≤5 versus >5 plaque-retentive factors in the OPG** | 4.0  versus  2.7 (p<0.001) | 4.4  versus  3.6  (p<0.001) | 4.0  versus  3.7  (p=0.041) | 4.4  versus  3.8  (p=0.002) |
